# Bored and spoiling for a flight: capabilities lost and found in lockdown

**DOI:** 10.3389/fsoc.2023.1215027

**Published:** 2023-11-28

**Authors:** Doris Sommer

**Affiliations:** ^1^Department of Romance Languages & Literatures, Harvard University, Cambridge, MA, United States; ^2^Department of African and African American Studies, Harvard University, Cambridge, MA, United States

**Keywords:** boredom, personal capabilities, lockdown conditions, human dynamism, personal and collective dysfunctions

## Abstract

Human beings are dynamic; our innate faculties beg to engage in activities. To achieve fullness and human dignity, people “convert” personal capabilities into active “functionings,” Amartya Sen explains. This means that staying still is not a normal state. It can feel like punishment. Forced inactivity will generate resentment, resistance, and boredom that can fester until pent-up energy explodes violently, or implodes in depression. Boredom defaults on capabilities and resources in many cases. In other cases, stillness is a gift. It can stimulate the imagination to fill in emptiness with memories and new explorations. Either boredom builds toward doing damage, or it releases energy to think and to create. What people don't do is stay put, mentally or physically. Authorities-including police, judges, teachers, parents –should take this dynamic human condition into account and reconsider the effects of conventional command and control policies. Then they can choose between violence and creativity as alternative outlets for the energy that boredom generates. Short of facing up to human dynamism, decision-making may continue to favor strong-arm tactics, which trigger the violence and pain that policing is meant to mitigate. Is it surprising that apparently peaceful peoplebecome enraged in lockdown conditions? Do adults wonder why students drop-out of school and suffer escalating rates of depression and suicide? Boredom is certainly not the only cause for these disastrous effects, but to ignore it risks remaining complicit with processes that perpetuate personal and collective dysfunctions. Complicity with harmful practices will miss opportunities to channel frustrated energy toward developing human capabilities. Authorities are responsible for promoting peaceful development. We are all responsible.[2] Normally, people stay busy with routine activities. We work, play, attend to family and to friends. Particular activities have even become our public badges of identity, as is evident in surnames (Cooper, Baker, Taylor, Farmer, etc.) that trace back to work that ancestors answered to. Lockdown during COVID-19 meant that many otherwise occupied people had few outlets for energy. Those who knew how to meditate managed to assuage anxiety through contemplation and the pursuit of ideal emptiness.

## Introduction

Human beings are dynamic; our innate faculties beg to engage in activities. To achieve fullness and human dignity, people “convert” personal capabilities (Sen, 1999) into active “functionings,” Amartya Sen explains^1^. This means that staying still is not a normal state. It can feel like punishment. Forced inactivity will generate resentment, resistance, and boredom that can fester until pent-up energy explodes violently, or implodes in depression. Boredom defaults on capabilities and resources in many cases. In other cases, stillness is a gift. It can stimulate the imagination to fill in emptiness with memories and new explorations. Either boredom builds toward doing damage, or it releases energy to think and to create. What people don't do is stay put, mentally or physically.

Authorities—including police, judges, teachers, parents—should take this dynamic human condition into account and reconsider the effects of conventional command and control policies. Then they can choose between violence and creativity as alternative outlets for the energy that boredom generates. Short of facing up to human dynamism, decision-making may continue to favor strong-arm tactics, which trigger the violence and pain that policing is meant to mitigate. Is it surprising that apparently peaceful people become enraged in lockdown conditions? Do adults wonder why students drop-out of school and suffer escalating rates of depression and suicide? Boredom is certainly not the only cause for these disastrous effects, but to ignore it risks remaining complicit with processes that perpetuate personal and collective dysfunctions. Complicity with harmful practices will miss opportunities to channel frustrated energy toward developing human capabilities. Authorities are responsible for promoting peaceful development. We are all responsible^2^.

## Lockdown laboratory

Normally, people stay busy with routine activities. We work, play, attend to family and to friends. Particular activities have even become our public badges of identity, as is evident in surnames (Cooper, Baker, Taylor, Farmer, etc.) that trace back to work that ancestors answered to. Lockdown during COVID-19 meant that many otherwise occupied people had few outlets for energy. Those who knew how to meditate managed to assuage anxiety through contemplation and the pursuit of ideal emptiness. But many mortals—including myself—either fail at efforts to concentrate on breathing, for starters, or they have not tried.^3^ Maybe people don't know about the disciplined techniques and the goal of inactivity; or maybe they are not drawn to doing what looks like doing nothing. Wanting to do something when it seemed that very little could be done prepared the conditions for widespread boredom. Households became laboratories for studying boredom, hothouses in fact, or petri dishes. The unbidden experiment is an opportunity and, therefore, an obligation to reflect on the dynamic and to expand a range of appropriate responses for future challenges. Either we will have learned something in the laboratory to help design a new paradigm for human energy under constraints, or we will consider the damaging effects of constraint mere anomalies in conventional, if disappointing, approaches to security, education, and mental health. To use Sen's term, we will “muff” the opportunity to develop.

When work and play activities ended abruptly in worldwide house arrest during the COVID-19 pandemic, human dynamism hit a wall, an interior wall of domestic confinement. The explosive effects of inactivity blew up beyond predictable proportions.^4^ Consider the spike in domestic violence as one unintended consequence of public health policy. Soon after the doors locked, alarming reports across countries and social classes repeated and confirmed statistics that seemed unbelievable.

Early on, “The World Health Organization has highlighted a 60% spike in calls to European domestic violence hotlines in April” (Mahase, 2020). Brazil's Public Health Forum registered an increase of 431% for the same period (through social media, given the danger of retaliation by eavesdropping partners) (Bueno et al., 2020, p. 13), while New York's Governor Cuomo appointed a Task Force to find innovative solutions for the sudden rise in home-based crimes, after reporting a 33% spike in April 2020 (New York State Task Force, 2020). Data have been also confirmed in a number of studies (African News Agency, 2020; Boserup et al., 2020; Bradbury-Jones and Isham, 2020; Campbell, 2020; Leslie and Wilson, 2020; Miltimore, 2020; Mlambo-Ngcuka, 2020; Usher et al., 2020; World Health Organization, 2020). Governments worldwide asked how to offset the aggressive repercussions of lockdown, according to The Council of Foreign Relations (Bettinger-López, 2020).

The question of what to do begs the question of why the lockdown increased domestic violence. Loss of jobs, alcohol, and psychological stress are still common and convincing answers (see footnote 22). Familiar responses to violence continue to be vital, including hotlines and shelters for victims and punishment for perpetrators. But neither the known causes nor the standard remedies managed to reduce the incidence of domestic violence even before the pandemic. By now, the limits of conventional approaches are evident. They amount to secondary or tertiary interventions, after threats are made or damage is already done. Something more is needed, urgently. It is primary prevention to stop violence before it starts (Walden and Wall, 2014). Prevention will include education about violence and newly learned practices that undo habits of power. In order to diffuse dangerous behaviors before they develop into aggression, we must first identify nonviolent but simmering conduct that can become explosive. Recognizing triggers for potentially violent behavior is a step toward proposing innovative remedies. One trigger is so evident that it has passed under the radar of many specialists and authorities: Boredom. People get bored in confinement. This is a hypothesis worth pursuing. Arguably, men may get more bored than women even under normal conditions (Gosline, 2007; Talbot, 2020; Westgate and Steidle, 2020). The effects of domestic boredom could turn out to be unremarkable, compared with the intense stresses of joblessness, poverty, and a bleak future. But this unexplored stressor is an opportunity for new and effective interventions to mitigate violence, even if they cannot end it or replace the need for economic security.

During a lockdown, when circulation stops between home and the outside world, the breakdown of normal activities and relationships can lead to anomie, an intolerable condition of normlessness, as Emile Durkheim defined it, when social definitions break down into chaos. In lockdown conditions, this collective disaster is often linked to personal, psychological, malaise that sociologists have identified in coercive institutional settings. Boredom. If feeling bored were a stable psychological condition it might be unfortunate for sufferers but not necessarily dangerous for others. In that case, there would be no reason to suspect it as a trigger for anomie and violence. But boredom is a state of simmering, volatile, energy. Numerous studies document the connection between boredom and violence, in penal centers, student residences, military or scientific encampments, and other spaces whose common denominator is the condition of total or partial, voluntary, or involuntary confinement. But little attention had been given to boredom at home, until the lockdown forced a reframing of this allegedly safe space into a laboratory for studying the general dangers of doing nothing and the remedy of turning empty time into a resource for creativity.

The psychological stress from boredom is today a major concern for children, whether or not authorities identify boredom as a stressor. Many young people were among the victims of physical violence at home, but practically all school-age children were themselves victims of boredom after being disconnected from real classrooms, even from virtual classrooms where the internet had failed to reach them or when it failed to connect. COVID exacerbated the loneliness and anxiety that had already become a mental health crisis before the pandemic. The lingering effects of lockdown amount to another pandemic, this time of loneliness, depression, and suicide among both youth and older people.

The COVID crisis came suddenly, and clinical responses were urgent. Understandably, attention focused on research about a disease that eluded the experts. All eyes were on trials of vaccines that could promise protection and that finally delivered some safety. But very little was done to mitigate the personal and social damages. Neither individual homes nor collective institutions got public support to adjust psychologically to the unforeseen corollaries of public health directives. Why was heightened violence a surprise? Should we not have expected it during a lockdown? One reason for the unpreparedness is that almost no one identified the menacing elephant of boredom that had installed itself in the locked down room.^5, 6^ The almost immobile beast didn't count as a character in the dramas recounted by domestic partners, or by state authorities, public health providers, or NGOs. The tragedies of domestic violence repeated familiar scripts of conflict between victims and perpetrators. Actors trudged toward pre-scripted conclusions: victims should be rescued and perpetrators punished. This stock casting was disastrously indifferent to lockdown conditions where the unacknowledged elephant took on a protagonist's dimensions while waiting for an author to notice, like a Pirandello character.[6] Other actors included those who suffered violence, but these players were no longer predictable victims whom standard script writers would evacuate to “safe houses.” Safe houses were anachronistic while women worried more about dying from the disease in women's shelters than about beatings from their partners. Would-be rescuers developed elaborate techniques for women under siege to contact friends or to alert the police. For what purpose? Forced isolation during COVID-19 practically vitiated the ingenious efforts. Where would the women go if they did manage to call for help, after risking more abuse from partners who eavesdropped and retaliated? Equally ineffective were the standard protocols of punishment for perpetrators. Police had cautions to consider, including their own safety from COVID. Under this sudden regime of restrictions, how could people count on courts and counselors, and incarceration? On another front, while schools stayed closed, how could children achieve an education to develop their capabilities as future contributors to economic and civic life? A filler for this gap was the viral spread of social media that accelerated beyond the already addictive pre-COVID levels. The result of lockdown was to further dismiss deliberation and to make false news indistinguishable from information, so that schooling continued to lose ground to AI shortcuts on isolating unipersonal devices.

The question of what to do begs the question of why the lockdown increased domestic violence. Loss of jobs, alcohol, and psychological stress are common and apparently compelling answers (see footnote 22). These familiar responses to violence can still make sense, if hotlines connect and safe houses shelter while perpetrators submit to control. But neither known causes nor standard remedies had managed to reduce the incidence of domestic violence even before the pandemic.^7^ By now, it should be clear that conventional approaches treat symptoms rather than causes. Therapy for violence responds to symptoms of damage already done. Prevention is in order (Walden and Wall, 2014) through unfamiliar proposals. To diffuse dangerous behaviors before they develop into aggression, it will be important to recognize triggers for potentially violent behavior.

One trigger is so common that it goes unremarked by specialists and authorities: Boredom. People get bored in confinement. And men, arguably, get more bored than women. This is a hypothesis worth pursuing. The results may turn out to be unremarkable compared to the intense stress of joblessness, poverty, and a bleak future. But the unexplored stressor is an opportunity to explore unfamiliar interventions that may mitigate depression and reduce violence. A lack of agility on the part of authorities exacerbated the COVID crisis, and the rigidity could keep us stuck in the failure of familiar practices.

## Paradigm shift

The failure is partly conceptual. It is a curable calcification that this essay addresses. My purpose is to explore better responses to current and future crises. A cure for staying stuck requires perseverance and patience along with imagination, Thomas Kuhn concluded from observing paradigm shifts in the history of science (Kuhn, 1962). Change takes an intentional will to shift from a familiar paradigm of cause and effect to alternatives (Kuhn, 2021). If experts stick to parameters of conventional thinking, the lockdown will have amounted to a mere pause from making sense, a blip, rather than a grant of time in a laboratory for new thinking. The shift in thinking about boredom begins by admitting that the conventions of keeping order simply don't work. Parents, teachers, or jailors impose conventional demands for obedience to everyone's peril.^8^ Potentially disruptive children and other wards may respond with a backlash that ignites the undesirable behavior that the demand for obedience had meant to control. This anomaly between command control and out of control responses is no mere interruption of rational expectations. It is evidence of a conceptual error, because people are normally dynamic. A correction can start by acknowledging the dynamism and supporting human capabilities. The difference between control and support amounts to a shift in paradigm, from obedience to autonomy, from being the subject of another's will to becoming a self-realizing agent.

This shift is part of a general overhaul in development studies. No longer persuaded by the conventional Gross Domestic or National Profit (GDP or GNP)—a single mathematical measure that ignores abysmal differences in living standards between rich and poor people^9^—the United Nations and other agencies now prefer a Human Development Index (HDI) of three criteria (longevity, literacy, income). Pakistani economist Mahbub ul Haq proposed the HDI to support people-centered policies^10^ The improvement came from a collaboration with Amartya Sen who had alerted economists to the difference between wellbeing and being well-off. Sen's “Capabilities Approach” to human development gives account of real people, their situated resources and their opportunities to use those resources (Sen, 1985). Use is key, Sen points out, to convert what people have into what they can do with what they have. Maybe peasants have crops but no transportation to sell them in markets, or citizens have the right to vote but no expectation that their votes will count. The distinction between assets and making good on assets depends on one's capacity to convert “being” into “doing.”^11^

Announced in 1974, this conceptual shift from a one-size-fits-all GDP to customized considerations resonated with a generation of feminists who were unmasking the male privilege and willful blindness of “universality” in law and economics^12^. “[T]he full human diversity among people is insufficiently acknowledged in many normative theories, such as theories of distributive justice. This also explains why the capability approach is often favorably regarded by feminist philosophers, or philosophers concerned with care and disability issues (e.g., Khader 2008, Terzi 2008, 2010),”^13^. Most notably, Martha Nussbaum took Sen's lead to multiply criteria for wellbeing by proposing a basic list of rights in order to identify actionable gaps that prevent people from “creating capabilities.^14^” But Sen demurs from lists: “What I am against is the fixing of a cemented list of capabilities. (Sen, 2004)” “There is nothing illegitimate or defeatist in recognizing that the valuation rankings of wellbeing may have gaps.^15^” While he concurs that we need bare and basic criteria, even helping ul Haq to devise the three-pronged HDI^16^, anything more, he argues, would miss the specific priorities and conditions of real lives.

In real life, certain conditions can seem intolerable to some people but not to others who share the same culture and social class. The lockdown is one condition that yielded dramatically different responses. In shared households, newly aggressive domestic partners became violent while others suffered the consequences. Some members of a household evidently experienced the lockdown as a breakdown of their normally benign behavior; their victims experienced the fallout. Aggression was largely one-sided, but the experience had two sides. What accounts for the difference between COVID's housebound victims and victimizers?

Where domestic violence is concerned, we know that a significant difference is gender (Peterman et al., 2020). Even when men don't start fights at home, they tend to finish them with force. Statistics on this age-old abuse had already scaled up alarmingly before COVID-19, maybe because increasing numbers of women were choosing to report crimes that would earlier have cost them economic security or would have seemed too commonplace to register. Improved reporting before lockdown put the spike in sharp perspective. What made so many more men violent during house arrest than before? It is worth considering a subjective differential for men and women in forced confinement.^17^

Though some people may actually enjoy the lull in inactivity to dedicate time to homey tasks, boredom burdens others to the point of intolerable rage. The feeling is subjective but the consequence is objective when one person's boredom explodes into violence toward others. This escalation from one to others is a secondary consequence of boredom and it is reason to spell out or to adjust Sen's capabilities approach. Perhaps an index of wellbeing should include interpersonal criteria. My attention to groups is different from communitarian arguments that can override a liberal focus on individuals. To favor collective cultural rights and norms, communitarians put group priorities above personal preferences. Sen rejects that favoritism. He defends the flexibility that personal preferences demand, to the point of refusing the arguments of even well-meaning list-makers like Nussbaum.

An interpersonal criterion like boredom for evaluating violence at home doesn't interfere with Sen's intentionally unfinished framework. Rather, it makes good on his defense of moving pieces. Consider the ways in which subjective desires and capabilities interact with shared consequences to recognize that setting priorities amid changing circumstances includes interpersonal dynamics. Personal criteria alone may miss some potentially dangerous dimensions of individual development. The danger lurks in Sen's focus on personal wellbeing, one human being at a time. “Thus narrowed, personal wellbeing related to one's own life will reflect one's standard of living^18^.” This self-possession is aspirational in some cases and unthinkable in others. Sen assumes that people can and should make reasonable and free choices about their identities and affiliations^19^, but the assumption is hardly reasonable in the real world of gender hierarchies and familial ties. This oversight may miss Sen's own cue about framing capabilities in collective contexts. Sen, like Nussbaum, stays with the individual, though he prefers improvisation in the volatile variety of contexts while she prefers more structure.

## Little women, big men

Does a conceptual framework centered on single persons allow a vestige of liberal universalism to persist in the capabilities approach? Universalism assumes that people are ideally equal and therefore interchangeable in theories of value and in measurement. An instance of this paradigm is the one-size-fits-all GDP that lingers beyond its expiration date. Despite being pronounced dead for decades, the single economic aggregate that obscures inequities doesn't quite die. A “zombie concept,” it haunts and hinders human development^20^. As a corrective to outdated paradigms of development, the capabilities approach proposed by Sen and pursued by Nussbaum adds criteria of measurements to a misleading aggregate (Sen, 2000; Nussbaum, 2000). By multiplying the criteria, they can focus on individuals rather than on whole nations. But even this deeper view risks missing an important dimension of development by blurring the interlocking forest with a focus on individual trees (Sen, 2001). “[W]e have responsibility for what we desire (and the need to relate it to what we value), whereas we have no such direct responsibility for the desire of others^21^.” Perhaps the operative word here is “direct,” because surely a culturally bound desire is a collective responsibility.

Gender-determined roles are zombie concepts too. Legal and economic discrimination by gender has been condemned for centuries and outlawed in more recent history, but the prejudices and practices of male superiority continue to skew access to rights and resources (Prager, 1996). Nancy Chodorow named the process of unequal gender formation “the reproduction of mothering (Chodorow, 1978).” One result of this formation is that women take charge of children and men achieve a paradoxical authority when children invest power in fathers as a safeguard against tyrannical mothers. Women rule at home. No wonder men can feel disabled when housebound. Surely they get more bored than women do during lockdown, since a consequence of gender specific training is that women know how to use domestic space to exercise agency, while men count on other spaces to convert their capabilities into functioning. Some readers may be skeptical about this gendered boredom quotient; they may also dismiss the relevance of boredom for making sense of explosive behavior. Those readers may also identify as male. Women don't doubt the gender difference or the threat, generally speaking. They recognize the syndrome of male listlessness, frustration, and rage^22^. Differential levels of male and female boredom offer a speculation worth pursuing in order to craft new practical responses.^23^

Nussbaum targets tenacious gender disparities as obstacles to women's development. By definition, disparities are comparative and therefore, interpersonal, a dimension that sometimes falls out of focus in Nussbaum's defense of autonomous selves, one woman at a time. To appreciate this slip from real and relational identity to ideal autonomy consider the example that launches Creating Capabilities (2011): “[A] small woman in her early thirties” Vasanati had abandoned an abusive husband and returned to her family of origin^24^. The example is telling beyond the conclusions that Nussbaum draws about Vasanti's courage and about women's solidarity through Sewa, a collective of working women. But Vasanti's brothers also played decisive transitional roles. As members of a Gujarati family, they were not obliged to take back a married woman, however abusive her husband became. When the brothers rescued their sister, their decision was unscripted. Their capabilities functioned to judge and to act. The brothers enabled Vasanti to become autonomous, first alone and then through Sewa. We might call this development a collective or a cascade conversion of family and community resources into a personal triumph that would bring more ripple effects. Nussbaum does give the brothers credit for valuing Vasanti's safety over community norms, but the narrative doesn't feature the structural connection between the woman's step forward and the men's accompaniment. Their advances were mutual. The brothers' decision enabled their sister to be free; and the sister's demand sparked her brothers' choice. Paradoxically, independence can depend on mutual support. But mutuality plays a minor role for Nussbaum. When she distinguishes between internal and combined capabilities, the difference refers to innate faculties and developed talents in an individual. Though they overlap, “innate equipment... into advanced capabilities” refers to the development of an individual^25^.

From her activist legal perspective and in Sen's ethical economics, the social element of wellbeing shows up, but mostly as a function of personal development. This is close to a contradiction since sociability is a structural connection between a self and others rather than a personal pastime. The focus on individuals means that social values or criteria for measuring development remains surprisingly underdeveloped. For both theorists, sociability as mutual care fades perilously; it flattens into a dimension of personal growth. This is a curious loss of depth perception, after both Sen and Nussbaum remind us that Adam Smith considered sociability to be a significant contributor to the collective Wealth of Nations^26^. In fact, Smith's earlier Moral Sentiment begins with mutuality, “an interest in the welfare of others, and make their happiness necessary to him, even if he gets nothing from it but the pleasure of seeing it^27^.” Perhaps understandably, given the tug today toward group-based recognition and authenticity, Sen and Nussbaum stay clear of communitarian claims. They put particular people first, and Sen acknowledges the incalculable variety of cultural resources that often include personally crafted hybrids of competing cultures. A capabilities approach that supports the autonomy of individuals understandably resists giving priority to culturally cozy traditions that can trump dynamic conversions^28^. Culture, in Sen's formulation, is not the end, or objective, of community life, but the means for development. It is a field for improvisational activity rather than a legacy to protect^29^.

Boredom is a personal experience that can flare into interpersonal trouble. It begins by oppressing an individual who cannot tolerate empty time. But when boredom builds toward violent interruptions of the tedium, victims are collateral damage. Shouldn't a boredom quotient figure among the criteria for wellbeing that Sen can enlist when appropriate? It is the elephant in the room of development theory, silently seething and preparing to pounce on an individual's reason and autonomy with consequent casualties nearby. Victims include battered partners, children, older parents, all of whom lose their own autonomy, resources, and agency for development, when lockdown is experienced as lockup. In wartime, this conversion of boredom into agency is linked to atrocities, and tedium is worth tracking in police brutality^30^. Conversions of energy into action are not always pro-social. This caveat to the capability approach points to a loose end in Sen's defense of pluralism regarding values. Sometimes, pathologies of power, revenge, violence will demand a more normative framework to distinguish abuse from empowerment (Wolff and De-Shalit, 2007).

To support human thriving, we have an opportunity (read obligation) to consider the interpersonal, social, dimension of capabilities that Adam Smith featured for personal wellbeing and that Sen updates: the opportunity is to promote in others the skills and the pleasures that support their wellbeing, and ours by extension. Perhaps this represents a double paradox. On the one hand, development is understood in the capabilities approach as self-development. Collective advances follow from exercises of personal autonomy. On the other hand, pleasure is often understood as a diversion from development, literally going off track. How can helping others to feel pleasure support one's own self-development? A short answer is that other people who don't feel pleasure are at risk of feeling rage. Self-interest will be well served by preempting other people's rage and the violence it ignites. Regarding ethical worries about pleasure, Sen and Smith relax the concern when they recover the link from pleasure to dignity and autonomy.^31^ “To lead a life without shame, to be able to visit and entertain one's friends, to keep track of what is going on and what others are talking about, and so on, requires a more expensive bundle of goods and services in a society that is generally richer^32^.”

Pleasure has had bad press since the philosophically hedonist heyday of British utilitarianism. Sen revives its ethical work. What would wellbeing mean without enjoyment? A standard of living makes sense “through some object of value—in this case, some type of pleasure^33^.” The reluctance to embrace an ethics of enjoyment and sociability has made pleasure a perverse pursuit. People seek it out, perhaps assuming it must come at a moral cost. Enjoyment seems tinged by sinful desire in a capitalist culture that devalues diversion and enshrines hard work. (Max Weber called this “an-hedonic” culture Calvinist)^34^. But pleasure and play have been ethical values at least since Aristotle and then for a long line of thinkers that extends, for example, through John Finnis, who included aesthetic experience as a dimension of personal development^35^.

Today the ethical difference between aesthetic pleasure and utilitarian desire-fulfillment may not be clear, but my admittedly simplified Kantian distinction will help to identify effective ways to deal with boredom. Aesthetics is social; desire is not. Free from material or functional interests, aesthetics is an experience of form rather than content; it depends on deliberation to process something surprising, unfamiliar, and still nameless. The surprise sparks a personal reflection that very soon becomes interpersonal to validate or to test an observation. Paradigms can shift in this process of disinterested experience and judgment. Desire, on the other hand, pursues an interest, something already known and named. It is driven by goals inside established paradigms. Disinterested delight is not a driver but a pause. The delight felt in aesthetic pleasure begins with confusion, not knowing what to think or to feel about something new. The activity of judgment stretches the effect of a new sensation through the enjoyment of deliberation with other people. Short of participating in aesthetic education, democracy cannot count on sociability and loses ground to personal desire.

## Art is therapy

Boredom is a lack of surprise, a death of sensation. Empty time can feel like deprivation rather than the luxury of leisure to think and to make things. For people who know how to use it, unprogrammed time is the resource of freedom to be converted into a functioning—in Sen's terms. But for bored people who resent empty time, the resource withers or flips into something monstrous. Boredom flattens freedom into frustration. Either a hiatus of activity will end in an engaging activity or in acting out. The tension that comes from inactivity is volatile, and the energy will come out one way or another. Since people have a range of innate faculties that can be converted into functionings, it makes sense to foster creative faculties. This is a matter of choice, arts over aggression. Skeptics may assume that choosing is limited to some people who are creative. Kant assumed this.^36^ That's why he featured judgment over creativity as the universal faculty for civic development. But the assumption about predetermined access to or exclusion from creativity condemns us to expect more spikes in violence and more depression and suicide. The threats of interpersonal and self-harm are likely to increase. We can anticipate continuing and recurring boredom from loss of jobs, resignation from jobs, confinement to refugee camps, prisons, failing schools, and possibly renewed house arrest.

Fortunately, people are innately creative, despite the skeptics. Creativity is hard-wired in the human condition. Our challenge is to recognize the resource and to make good on our innate faculties of imagination and judgment. These dimensions of being can be converted into skill-based doings to develop capabilities. It was Kant's disciple, Friedrich Schiller, who gave this natural resource a name, Spieltrieb, playdrive.^37^ During the Terror of the French Revolution, Schiller elevated this faculty to the level of a drive to interrupt the spirals of violence that had locked the other two drives—Reason and Passion—into mutually murderous opposition. How do you interrupt the escalating, self-destructive, spirals? Schiller's answer was to bridge the feuding forces through play and art. Anyone can play, and we can learn to make art that will repurpose energies and divert the attention of enemies from hatred to delight in new forms^38^. Rage today includes flares from the counterintuitive trigger of doing nothing. Whether despair and death follow from revolution or from being bored, Schiller would urge us to take a step back and redirect our energies from aggression to artmaking. There is really no alternative, because without the “disconnection” from habits and interests that art provokes, people lose agency and stay caught in spiraling structures^39^. Convention may shun the arts as impractical in dire conflict, but Schiller knew that it was the missing agent of reconciliation^40^. Without art, conflict has no escape route. The opposite of play is not work or seriousness, anthropologist Gregory Bateson would explain for socio-ecological reasons;^41^ it is boring one-dimensionality that leads a species to extinction^42^.

## Symbolic destruction

Psychoanalyst Donald Woods Winnicott would come to Schiller's same conclusion. We have no evidence that Winnicott read Letters on Aesthetic Education (1794) (see foot note 52), but he was equally dedicated to play^43^. Schiller had written “man is truly human when he plays, and he plays when he is truly human,” as if presaging Winnicott's work^44^. The therapist included his own practice among creative activities: “Psychotherapy takes place in the overlap of two areas of playing, that of the patient and that of the therapist. Psychotherapy has to do with two people playing together^45^.” This means free and “non-purposive” artmaking^46^.

The instinct to create shows up immediately, says Winnicott, when a newborn searches for its mother's breast^47^. The breast materializes because the mother plays too, bending to the baby's will to welcome it as creator of its world. The early games multiply throughout life as “play is the continuous evidence of creativity, which means aliveness (Abrams and Hjulmand, 1996).” People play at affecting the world, not only in response to hostility or to loss, as Melanie Klein thought.^48, 49^ Creativity is an innate drive—Schiller's playdrive—to achieve tacit control over existing, often conflicting, materials and demands, as when a fifth grader cures her anxiety about math by drawing scary but funny comic strips. In professional “Newspeak” this recourse to pleasure is called “self-administered art therapy.”^50^ We can call it play. Riskiness spikes both art and analysis with dangers of unpredictability, dangers that cannot be abolished if therapy is to proceed. So, the work demands a steadiness that can anticipate and survive aggressive surprises meant to unhinge the playmate, including the clinician^51^. “The drive is potentially ‘destructive'... But destruction of an object that survives, that has not reacted or disappeared, leads on to use (Winnicott, 1969).” And using people, Barbara Johnson understood after reading Winnicott, amounts to loving them (Johnson, 2000).

A nadir of activity and a peak of interaction made for a perfect domestic dust storm during lockdown. Reduced access to psychologists and psychoanalysts, the lack of money to pay them even when therapists were available on electronic platforms, and the failure to focus on the elephant of boredom in the room all converged to heighten domestic violence during lockdown. Already elevated statistics that spiked during house arrest should now prompt us to recognize an antidote to the malady of boredom. It is the friendlier face of the elephant. If boredom is a common and unacknowledged threat, art making is a common but underemployed resource. Rising levels of anxiety, depression, abuse, and suicide prompt mental health providers to explore support from non-clinical practices. Are there effective resources outside the clinic? There are. In fact, a social approach to wellbeing is well known, and new pioneer programs train lay counselors and peer mentors to offer support.^52^ These resources could substantially expand and deepen by adding the under-explored option to engage artists as allies.

## Stigma

Low resourced settings are particularly fertile for this option because poor areas are often rich in arts. Traditions of storytelling, music, dance, weaving, cooking, mask making, poetry, pottery, etc. still thrive in everyday activities alongside modern practices. Artist Pedro Reyes notes that Latin America, for example, may be poor in finances and in politics, but in art it is a treasure house. This can be said of the global south in general. Consider an example from Peru's highlands. Peasant women there cultivate up to 4,000 varieties of mostly inedible potatoes, just for their color and shape, to lay out designs for new weavings.^53^ The challenge for therapy in economically poor environments—and everywhere else—is to dignify art as a worthy partner for therapy.^54^

Stigma has been an obstacle. It is an equal opportunity blight. On one side, people with mental illness don't seek help when stigma stops them; on the other side, providers often present a stigma against taking art seriously. They tend to dismiss creativity as unscientific, even risky, and inappropriate. The arts seem either inconsequential or downright harmful. While creativity is no sure pathway to mental health, and though artists may continue to be afflicted by the demons that drive them, it is worth considering the differing degrees of affliction when art is an outlet and when it is not. Memoirs offer testimony to the power of creativity as a coping mechanism for mental illness.^55, 56^ But clinicians and researchers have been reluctant to credit artmaking as therapy, as a medium for amelioration, even when the making seems meaningless.^57^ Art is therapy. The reason is clear. Artmaking is active. To create something new demands dynamic faculties of mind (Stuckey and Nobel, 2010). When people make art, they become observers, exercise judgment, experience autonomy, make decisions, and anticipate communication through visual or performative languages, including words repurposed for poetry and prose.^58^ To assume that art works because it makes meaning is to miss the magic of making itself.^59^ Art works because it wedges a distance between what is and what could be. Augusto Boal^60^–founder of Forum Theater and author of Theater of the Oppressed (Boal, 2000) put it this way:

“Only the human being is tri-dimensional (the I who observes, the I-*in-situ* and the not-I) because it alone is capable of dichotomy (seeing itself seeing). And as it places itself inside and outside its situation, actually there, potentially here, it needs to symbolize that distance which separates space and divides time, the distance from ‘I am' to ‘I can be', and from present to future; it needs to symbolize this potential, to create symbols which occupy the space of what is, but does not exist concretely, of what is possible…So, it creates symbolic languages: painting, music, words.”^61^

Rational choice theorist Jon Elster counted on artists to illustrate good decision making, because artists know how to limit options and manage constraints (Elster, 2000). This cluster of mental and emotional talents can channel explosive energies into “symbolic destruction,” Winnicott's name for artmaking (Winnicott, 1991). The dichotomy that Boal identifies between being and observing can crack fissures in lockdown states of anxiety and depression, to let the outside world slip in. At a mental hospital in Paris, Boal discovered that on stage, when performing for other patients and their doctors, even acutely afflicted people would budge beyond their static clinical narratives. On stage, patients become actors to be observed by themselves and by others. Both actors and spectators occupy a fictional space that distances and estranges habitual stories which—from this unfamiliar perspective—can be told differently. After acting, sessions with therapists went better at the hospital. Aesthetic space was the opening.^62^

Artists are not victims. They are neither stuck in one-track narratives nor stopped by debilitating circumstances. Instead, artists are agents who transform problems into challenges; they recycle found materials into new products. Whether or not the products have aesthetic value for connoisseurs, the therapeutic effect of artmaking comes from the process, often with byproducts of pride in the work and in oneself.^63^ Sometimes artmaking reveals buried trauma that a talking cure doesn't easily access. This contribution has attracted the sleuthing attention of psychoanalysts.^64^ But other times, artmaking is untethered to repressed meanings. It simply exercises freedom to explore materials and their arrangements. Artmaking can be a purposeless exercise of agency and choice, a self-authorizing therapy against lethargy and listlessness.

Even unhappy people, like Boal's hospitalized actors, will accept an invitation to play, though they may bristle and retreat from unfairly demanding prompts given by many clinicians to find meaning or purpose.^65^ The invitation to participate in artmaking can be as unassuming as making a meal or getting dressed, putting together pieces of clothing to create an outfit. Arts may include drawing a picture or singing. (The Spanish verb interpretar for making music captures the inevitable innovation through performance.) Other, more intentional, traditional, or international, art practices can follow and develop or morph over a lifetime. The everyday nature of low-stakes artmaking sidesteps the stigma that would single out mentally distressed people for special attention. This mitigating feature of inconspicuous inclusion emerged in a pilot program for ninth graders in the Kibera slum of Nairobi where roughly half the youth are clinically depressed. The arts-literacy pilot did not single out mentally ill classmates, since all participated. The simple prompt to make art from a required text charmed even reluctant readers to create liberating interpretations, through drawings, rap routines, riddles, dance, theater, and other art forms of their choice. One result was a significant reduction in depression and anxiety. Another was enhanced pleasure in reading.^66^ Parallel projects in a Buenos Aires mental hospital confirm the findings (see footnote 82).

Genres and styles of artmaking will vary among regions and even between households, but the practice of making new things and performances from available materials and conditions is hard-wired in human beings. “Art making is an innate human tendency, so much so it has been argued that, like speech and tool making, this activity could be used to define our species (Dissanayake, 1992).” If artmaking is a human birthright, as philosophers and educators have long noted, we can count on a creative mental faculty to address boredom. We can also count on an abundant roster of mentors to channel energy into artmaking. Artists are everywhere. They can rise to the challenge of what to do with unbidden free time. Should we consider free time to be an inevitable trigger for trouble and prepare more prisons? Or can it be a resource for the conversion of capabilities into functions?

“There is nothing either good or bad, but thinking makes it so,”^67^ Shakespeare quipped. Interpretation makes the capabilities approach work in unscripted ways. Prepared scripts of behavior can “muff” genuine advantages, Sen observes. ^68^Free time, one of those unacknowledged advantages, is muffed when bored people feel helpless without prescribed activities. Policy makers may be equally stuck in predetermined scripts. Sen counts on people's innate reasonableness to take advantage of opportunities. But Shakespeare's lesson about interpretation adds creativity to reasonableness. The addition turns information into something new. In fact, Sen defends creativity as a dimension of reason (Sen, 2012). He stretches Bentham's identification of material and mental satisfaction to leave room for “creative discontent and constructive dissatisfaction.”^69^ Creativity is born of dissatisfaction. New ideas and things respond to “what is missing.” While Bentham's brand of happiness depended on desire-fulfillment, Sen warns that this goal will short-change capabilities if poor or oppressed people adjust to desires within reach.^70^ His update of utilitarian philosophy, elaborated by commentators and critics, points out that desires are not always constructive. Sometimes they are antisocial, like interrupting intolerable boredom by inflicting pain on others. Fulfilling a personal desire can come at a high cost to others.^71^ Can we provide creative programs that embrace freedom rather than fear it?

Sen's exhortation to be flexible about culturally conditioned values^72^ raises an ethical challenge: Should a community always support the conversion of personal capabilities into functions? Should we assign a positive value, for example, to the conversion of unbearable boredom into violence? How should policy respond to this danger in Sen's non-normative thinking? Nussbaum answers with a list of fundamental norms to respect. But Sen's non-normative lead takes us in a different direction. It would recognize the danger of violence as an incentive to prepare alternative pathways from boredom to self-expression. The alternatives to physical and verbal violence could be the varied forms of “symbolic violence” called art. A non-normative approach to thriving need not restrict or reprimand aggressive energies. Instead, energies can be channeled into pleasant, even passionate activities. A range of non-destructive activities could add up to a framework for redesigning policies of violence prevention. The utilitarian tone of this lead recalls Jeremy Bentham's brand of hedonism, to increase pleasure and happiness for the greatest number of people (Bentham, 2003). Sen notes that by now, the word “utility” has been hijacked by economists who use it to mean maximization of resources, whether or not this fulfillment of market-driven desire leads to greater happiness. “Mathematical exactness of formulation has proceeded hand in hand with remarkable inexactness of content.”^73^

Levels of boredom will not figure in GDP calculations, based on national aggregates that fudge glaring economic inequalities.^74^ But the capabilities approach can acknowledge boredom as a variable for wellbeing.^75^ If boredom were considered a resource of time and freedom, providing creative activities would help people use what they have to do what they can. Sen illustrates the difference between having and doing with the example of a bicycle: Someone who knows how to ride, turns the object into a resource, whereas someone else does not.^76^ The process is “equally concerned with the conversion of more intangible resources, such as human capital and public goods (Nussbaum et al., 1993).” Training in creative arts, for example, turns free time into a resource. The conversion begins with a choice of perspective: To see the menacing elephant in the room as a platform for freedom. “There is nothing either good or bad, but thinking makes it so.” If the capabilities approach is best understood “as a ‘thin' framework, which can be filled in by ‘thicker' theories and applications (Qizilbash, 2012),” why not fill in with the right to be creative, the Spieltrieb, our most characteristically human capacity (von Schiller, 2015).

Now is the time to notice an opportunity to adopt a fuller paradigm of functionings through programs to be developed by public and private institutions. The programs will feature more creativity and less control.

Under lockdown, there were mitigating circumstances that delayed this now urgent moment of stock taking. The rush of first responses to COVID's rising levels of infection and fatality kept us fixed on daily statistics, detailed by country, city, and neighborhood, while corollary crises of inequities of food supply and services intensified. Violence at home and in public, dropouts from school and from work, despair among young and old, are familiar crises that demand sharper focus than we have given. Perhaps there is a systemic malfunction in societies that value competition over collaboration, consumption over creativity. After the urgency to develop and distribute COVID-19 vaccines abated, a respite from immediate clinical questions is time to face the horrors of interpersonal and self-inflicted violence, suicides, massacres, police abuse. A paradigm shift is in order.^77^

## Connect the dots: desire and capability

Had legal and healthcare agencies considered a paradigm shift during lockdown, away from the familiar formula of staying still, masked, isolated, and controlled, had they dared to defamiliarize inactivity and noticed that it allowed an elephant to occupy the room, policy might have improvised creative programs. The lens of human capabilities might have re-framed the crushing beast of boredom into an enabling companion for play.^78^ Play can convert faculties into capabilities. The hiatus in conversion activities didn't figure among the causes that experts linked to the spike in domestic violence. Instead, they pointed to increased levels of alcoholism and drug abuse. Of course addictions increased, but this finding doesn't address root causes or possible remedies. Arguing that drunkenness and drugs were causes of violence is like observing that suicide is caused by depression without asking why a person is depressed. What caused higher consumption of drink and drugs? Observers duly reasoned that it was the loss of jobs and social isolation. Does that make addiction inevitable and domestic violence unavoidable under conditions of lockdown? Financial hardship and lack of social contact were indeed conditions of lockdown, while work and play were on hold. But these constraints demand creative policies to mitigate the menace of violence. Otherwise, expert observations about joblessness and addiction would suggest that domestic violence is a tragic but necessary price to pay for the clinical advice to stay home. Nothing to be done about it.

Reframing what seems inevitable is what art can do. Without art, habit shrouds our perceptions under the indifferent gray of what we already know. Boredom inhabits this gray zombie existence, haunted by colorless habit until it triggers a red rage. The gray went unremarked. Normal, familiar, and apparently unworthy of scholarly or activist attention, boredom was too obvious to talk about. It is the unspeakable elephant in the room, not because the beast is too frightful to confront but because it didn't seem worth talking about.[78]

## Lockdown countdown

A group of Brazilian professional soccer players took note. They turned their game into a resource for violence prevention. Amigos do Bem had formed years earlier to provide sports training, food, and other services to underserved young people and their families. They channel the national love of soccer into love for people and support for their development. With the pandemic, the players created Futebol Viral, a creative program in primary prevention of domestic violence. With good humor and enviable skill, Amigos do Bem directed men's desire for action toward repurposing the home as a field for sports. Futebol Viral engaged men in manly sports-based activities while they were stuck in tight quarters. Given the urgency of the moment and the statistics of domestic violence, the program postponed the players' long-term goal to reform gender assignments while they acknowledged existing contrasts between men and women. Surprisingly for some of us, soccer is as popular among women in Brazil as among men. The difference between them is the range of activities each gender pursued during confinement. Men were at a greater loss than were women, and the violence prevention program consisted of activities that men could enjoy. This was a clear case of the interpersonal conversion of capabilities into human development. Women's wellbeing depended on men's skills to enhance their own happiness. Futebol Viral has a family resemblance to many on-line arts activities that stimulated participation during the pandemic. Orchestras, choirs, dance companies, conversations, proliferated to fill in the time, but also to take advantage of it. The soccer option also took advantage of men's preferences, since few were drawn to more elite varieties of programming. But all these creative activities are options and inspirations for imagining a general paradigm for violence prevention.

Primary prevention is a public health strategy that includes reducing risk factors (Celentano and Szklo, 2019). Although prevention was originally designed to avert disease, this strategy has become essential to mitigate risk factors related to violence against women, now that domestic violence is recognized as impacting physical health, mental health, and social wellbeing (Walden and Wall, 2014). Among the risk factors for violence against women, studies recognize gender inequality, social norms and practices that condone violence, weak sanctions, and insufficient resources for support (Flood and Webster, 2007). Still lacking are the resources for dealing with boredom. This is a significant concern for public health policies that aim to prevent pathologies. Rather than “simply accepting or reacting to violence, its starting point is the strong belief that violent behavior and its causes can be avoided” (World Health Organization, 2002, p. 45). Boredom is one of the causes yet to be featured in recommendations for redress of blindness to causes of violence.

Men get bored at home and boredom made them more violent during lockdown. Conventional gender roles still structure many families and make the home the space of engagement for women, and disengagement for men. The challenge was to develop enjoyable activities for men at home. Soccer, for example. How does one translate (literally to move from one place to another) some of the pleasures of the open-air playing field to home based activities? Amigos do Bem got busy improvising answers. On readily available communications platforms such as WhatsApp and Facebook, technology helped to move soccer from the field to the house where offers of exercise, lessons in fancy footwork, live webinars, video games, book clubs, and art making multiplied the pleasures of being at home. Boredom could be banished from domestic life. During the lockdown, the WHO discovered soccer too. It announced an alliance with FIFA, to engage the world of soccer in a campaign against domestic violence (Al Jazeera News, 2020). To what extent can enjoyable soccer-related experiences mitigate this risk factor for violence at home? It was a practical question about reducing violence by mitigating boredom.

If boredom is the absence of stimulation, logically stimulation and activity can be remedies. But the logic is paradoxical for some people. As an intervention against gender violence, two obstacles arise: (1) Pleasure carries the stigma of perversion or danger, as if it were a detour from the obligation to improve ourselves. (2) Violent or potentially violent men seem unworthy as beneficiaries of pleasurable programs, even if these constitute an approach to primary prevention against male violence. This objection was voiced by the Secretary of Civic Culture in Bogotá Colombia. [79] It is precisely this inhibition of pleasurable activity that causes boredom among disengaged men. The paradox of proffering pleasure to perpetrators is not a contradiction but an unexpected connection. A dispassionate consideration of available tactics to curb harmful behaviors at home will have to confront a stigma against pleasure and cure it, because the home will not be safe until it becomes a pleasant environment for everyone. Not to take the cure is to rehearse conventional scripts about violent men and vulnerable women as we watch, with alarm, the rise in cases of domestic violence and depression.

The anhedonia of boredom can be cured by pleasure if this value of wellbeing is recovered. In its current and prolonged state, restrictions on pleasure are avenged in violent versions of pleasure-seeking. The Futebol Viral project posed two main questions: (1) Can the experience of soccer at home reduce boredom and help to make the home a pleasant space for men? (2) Will this resignification of domestic space lead to a mitigation of domestic violence? The confinement caused by COVID-19 made it the ideal time to focus on the chronic problem. More glaringly than ever, we noted the lack of research on the relationship between domestic boredom and domestic violence. Numerous studies document the connection between boredom and violence. They study penal centers, student residences, military or scientific encampments, and other spaces whose common denominator is the condition of total or partial, voluntary or involuntary confinement. There is no study about boredom at home. Having identified this knowledge gap, we understand that a basic research study is needed to answer the above questions since they have not yet been addressed through empirical data. For this, it will be necessary to carry out a quantitative study based on interviews with those who can testify to their experiences of boredom, violence, and pleasure during COVID19. See https://culturalagents.org/futebol-viral/.

## Pre-texts as childs-play

For children during lockdown, boredom was a bane of their diminished lives. The effects of missing school, friends, and activities were almost immediate. And the after-effects show high levels of depression and despair worldwide. Psychological wellbeing has by now become an earnest concern for many public and private agencies. Though Amartya Sen had not taken mental health into serious account when he began to develop his capabilities approach, it soon became an important horizon for him.^79^

After the lockdown had lasted for months with no promise of letting up, a desperately unhappy 8 year old in Buenos Aires prompted her worried mother to create a customized activity for her daughter. Tálata Rodríguez cleverly converted Pre-Texts, an arts-based literacy program, into a virtual practice for touchless times. She recruited a few parents of Eva's classmates to initiate a WhatsApp group for ten isolated fourth graders.[81] Again, personal wellbeing for the girl would depend on the happiness of others. Tálata is a poet who began her work with Pre-Texts in a 2018 collaboration between Cultural Agents Inc. and the City Housing Authority (IVC). The objective was to ease a massive relocation campaign. Few people wanted to move from their homes, however miserable the structures seemed to outsiders. The arts-literacy campaign helped to encourage slum dwellers to move from their cozy but unsafe housing to more sturdy buildings in bright but unfamiliar neighborhoods. Literacy, we know, is as important an indicator as longevity and income for the “Human Development Index.” And Pre-Texts makes literacy fun. The international program has lasted in Buenos Aires through repeated municipal grants that confirm the efficacy of arts-literacy to develop capabilities among at-risk youth. See www.pre-texts.org

Before Pre-Texts became a standard practice on zoom during lockdown before it was adapted as a mental health intervention in Nairobi, Tálata's WhatsApp pilot had demonstrated how to support wellbeing through improvisation under apparently paralyzing constraints. This is a case of achieving advanced functions by engaging capabilities in an otherwise desperate environment. Schools were at a loss. Teachers lost students to a lack of laptops and breakdowns in zoom. Childish joy withered. Tálata improvised. Where there was no internet, there was phone service. Rather than stress over access to zoom which favored the haves and excluded the have-nots, WhatsApp was available to all. On the hand-held platform, the Pre-Texts protocol adjusted to limitations. Instead of reading aloud to an assembled group of students, the way we normally begin Pre-Texts (imitating the “lectores” in tobacco factories), under lockdown one student recorded the text that all the students were required to read for school. Then he uploaded the recording for the WhatsApp group so that the others could recover it. In the next step, when each student asks the text a question and hangs it on a clothesline, the new version with isolated participants collected questions on WhatsApp. Then the participants posted answers; they took photos and pasted their drawings; they proposed new activities and continued to “play” with the assigned text. In this asynchronous but collective platform, the children regained agency and social contact. Even better, their reading and writing improved despite the isolation, as they converted lessons into playthings and learning into shared fun.

## A caution against caution

Transforming lessons into play may sound naughty or daring, unless we remember that “school” (σχoλ$\overset{´}{\eta }$ scholē) means leisure in both ancient and modern Greek. School is the time and space for exploration and discovery. So, the playful paradigm shift is a paradoxical recovery of ancient wisdom that recognized the dignity of pleasure. Paradigm shifts feel risky. Like moving from cozy dank slums to airy new neighborhoods, switching from outworn assumptions to new ideas raises resistance even when the novelty promises improvements. Take the familiarity of domestic settings for example, where boredom looms like an elephant. Clearly, the threat of domestic violence demands consideration of both the dangers and the mitigating opportunities. But there has been little consideration of boredom at home by authorities, health professionals, or policymakers. People tend to ignore boredom. The issue itself sounds tedious rather than triggering. Even for those who can be persuaded to perceive the rumbling threat and to consider the creative cure, more familiar and less effective thoughts about idleness and arts may survive the new knowledge. Zombie paradigms haunt us. One such zombie concept is that art is decorative and pleasant, but not necessary for wellbeing. In fact, art should be understood as a convenient name for change itself. How can change happen without imagining counter-factual and surprising alternatives to what already exists?^80^ Considered to be decoration or a luxury, art has been narrowly understood as a product of culture rather than as the dynamic process of making. Better understood as a “verb” than a “noun,” art is something intentionally made to interrupt the boredom of habit and to revive care for the world. Art is artifice, in Viktor Shklovsky's elegant formulation.^81^ The simple word raises difficulties in European languages because art, like culture, has two incommensurable meanings: the decorative or collectible product vs. the exploratory process; the shared patrimony of practices and beliefs vs. the creative interruption of that patrimony.^82^

Change requires interruption. It is an art of improvisation, risk-taking and judgment. But policy makers usually prefer a sure bet. Perhaps this is one reason for overlooking art as a resource for development. Art takes risks. If culture means only a legacy of shared sites and beliefs that bond communities, the bridging capacity is forfeited. Making new artistic projects can bridge diverse communities and foster social capital through the conversion of personal capabilities into enhanced social functionings and collaboration. For decision makers who manage to make the shift from considering only the GNP as the index of development to adopting a capabilities approach, the corollary will be to promote wellbeing through behavioral changes. In the spirit of Amartya Sen, these changes will value personal autonomy and self-fulfillment while taking local conditions and preferences into account. It is a creative improvisation, “a simultaneous and two-way relationship between functionings and capabilities.”^83^

Like art, development takes reasonable risks to change perceptions and expectations. Making something new with existing resources and constraints converts capabilities into skills. Art is the process of conversion from what is to what can be. We can count on an inexhaustible human resource for this process: creativity, the play drive, a fuel that policymakers can count on. With artmaking, free time is a gift rather than a threat.

The paradigm shift in policy will be to invest in participatory art projects rather than in prisons and police. For authorities in city government, schools, prisons, hospitals, etc., participatory artmaking can address otherwise daunting challenges, including security, education, public health, and immigration. The low-cost, high-impact projects that resolve boredom into skill building through youth orchestras, mural crews, dance troupes, theater companies were well-known but under-exploited before the pandemic. This user-friendly approach continues to be available and to be discounted. It is the therapeutic view of a ubiquitous elephant, an unhurried companion for leisure activities who occupies the same space as the brooding beast of boredom. Perhaps the laboratory experience of lockdown can yield an important if redundant finding: People are dynamic and need outlets to channel potentially explosive energy into collective arts. A paradigm shift from policing to participation will be an advance in human development for each one of us and for our interlocking societies.^84, 85^

## Data availability statement

The original contributions presented in the study are included in the article/supplementary material, further inquiries can be directed to the corresponding author.

## Ethics statement

Ethical approval was not required for the study involving humans in accordance with the local legislation and institutional requirements. Written informed consent to participate in this study was not required from the participants or the participants' legal guardians/next of kin in accordance with the national legislation and the institutional requirements.

## Author contributions

The author confirms being the sole contributor of this work and has approved it for publication.

## Footnotes

^1^Stanford Encyc: “(Sen, 1992: 39). To say that functionings are constitutive of a person's being means that one cannot be a human being without having at least a range of functionings: they make the lives of human beings both *lives* (in contrast to the existence of innate objects) and also *human* (in contrast to the lives of trees or tigers).”

^2^Stanford Encyclopedia: “Nussbaum passionately advocates that all people all over the world should be entitled, as a matter of justice, to threshold levels of all the capabilities on her list; but apart from mentioning that it is the governments' duties to guarantee these entitlements (2006; p. 70), she remains silent on the question who precisely should bear the burdens and responsibilities for realizing these capabilities. Yet as O'Neill (1996: chapter 5) has argued, questions of obligations and responsibilities should be central to any account of justice.”

^3^For more information on the benefits of meditation during lockdowns and stressful situations, see: Sharma, M., and Rush, S. E. (2014). Mindfulness-Based Stress Reduction as a Stress Management Intervention for Healthy Individuals. Journal of Evidence-Based Complementary and Alternative Medicine, 19(4), 271–286. https://doi.org/10.1177/2156587214543143

^4^For a comprehensive overview of the impact of lockdown measures on mental health and well-being, refer to: Brooks, S. K., Webster, R. K., Smith, L. E., Woodland, L., Wessely, S., Greenberg, N., and Rubin, G. J. (2020). The psychological impact of quarantine and how to reduce it: rapid review of the evidence. The Lancet, 395(10227), 912–920. https://doi.org/10.1016/S0140-6736(20)30460-8

^5^Dr. Bruce Price, Head of Neurology at Mass General and Director of Harvard's Center for Law, Brain, and Behavior, offered this characterization of boredom as the elephant in the room.

^6^For a study on boredom during the pandemic and its consequences, consider: Boylan, J., Seli, P., Scholer, A. A., and Danckert, J. (2021). Boredom in the COVID-19 pandemic: Trait boredom proneness, the desire to act, and rule-breaking. Personality and Individual Differences, 171, 110387–110387. https://doi.org/10.1016/j.paid.2020.110387

^7^For a detailed examination of the factors contributing to domestic violence during the pandemic, refer to: Peterman, A., Potts, A., O'Donnell, M., Thompson, K., Shah, N., Oertelt-Prigione, S., and Nicole van Gelder. (2020). Pandemics and Violence Against Women and Children. IDEAS Working Paper Series from RePEc.

^8^For a philosophical exploration of boredom, its nature, and how it affects individuals and society, see: Svendsen, L. F. H. (2005). A philosophy of boredom. Reaktion Books.

^9^A comprehensive analysis of alternative measures of economic performance and social progress beyond GDP and GNP, is provided in Stiglitz, J. E., Sen, A., and Fitoussi, J.-P. (2010). Report by the commission on the measurement of economic performance and social progress. Statisztikai szemle, 88(3), 305–321.

^10^Haq. (1996). Reflections on human development: how the focus of development economics shifted from national income accounting to people-centred policies, told by one of the chief architects of the new paradigm (p. xix+252–xix+252). Oxford University Press. See also *https://hdr.undp.org/data-center/human-development-index#/indicies/HDI*.

^11^Amartya Sen, 1974, “Informational Bases of Alternative Welfare Approaches: Aggregation and Income Distribution”, *Journal of Public Economics*, 3(4): 387–403. He clinched the argument in the Tanner Lectures at Stanford in 1979 *https://www.ophi.org.uk/wp-content/uploads/Sen-1979_Equality-of-What.pdf*, later published as *The Standard of Living*, 1986.

^12^Amartya Sen, 1974, “Informational Bases of Alternative Welfare Approaches: Aggregation and Income Distribution”, *Journal of Public Economics*, 3(4): 387–403. He clinched the argument in the Tanner Lectures at Stanford in 1979 *https://www.ophi.org.uk/wp-content/uploads/Sen-1979_Equality-of-What.pdf*, later published as *The Standard of Living*, 1986.

^13^Stanford Encyclopedia capabilities Approach.

^14^“Capability and Well-being”, in Nussbaum and Sen (eds.), *The Quality of Life*, Oxford: Clarendon Press, 1993) pp. 30–53. See also Martha Nussbaum, *Creating Capabilities: The Human Development Approach* (Harvard UP 2011).

^15^Sen, *Commodities*, “The tyranny of ‘required completeness' has had a disastrous effect on many other problems in economic measurement. . .. offering us a false choice between silence and babbling. . .. It is important to recognize that many economic and social relations are inherently partial and incomplete. . .” p. 31. Amartya Sen (2004) Capabilities, Lists, and Public Reason: Continuing the Conversation, *Feminist Economics*, 10:3, 77-80. https://doi.org/10.1080/1354570042000315163

^16^Sen, 2004, “Capabilities, Lists. .. “when my friend Mahbub ul Haq asked me, in 1989, to work with him on indicators of human development, and in particular to help develop a general index for global assessment and critique, it was clear to me that we were involved in a particular exercise of specific relevance. So the “human development index” (the HDI) was based on a very minimal listing of capabilities, with a particular focus on getting at a minimally basic quality of life, calculable from available statistics, in a way that the GNP or GDP failed to capture.” P. 79.

^17^Piquero, A. R., Jennings, W. G., Jemison, E., Kaukinen, C., and Knaul, F. M. (2021). Domestic violence during the COVID-19 pandemic - Evidence from a systematic review and meta-analysis. Journal of Criminal Justice, 74, 101806–101806. https://doi.org/10.1016/j.jcrimjus.2021.101806 Highlight the importance of addressing gendered dynamics in efforts to prevent domestic violence during the pandemic.

^18^Sen *Standard*. . “At the risk of over-simplification, it may be said that we move from agency-achievement to personal wellbeing by narrowing the focus of attention through ignoring “commitments,” and we move from personal well-being to the standard of living by further narrowing the focus through ignoring “sympathies” (and of course “antipathies,” and other influences on one's well-being from *outside* one's own life).” 38, 37.

^19^Sen, *Identity and Violence: The Illusion of Destiny*, “A person may decide to seek closeness with more than one of these predefined cultures or, just as plausibly, with none. Also, a person may well decide that her ethnic or cultural identity is less important to her than, say, her political convictions, or her professional commitments, or her literary persuasions. It is a choice for her to make.” p. 154.

^20^Eduardo Gudynas “Buen vivir: Today's tomorrow” “Critical positions that counter the myth of development have been repeated several times over the last 40 years. There are countless reactions from social movements against the negative effects (both social and ecological) of many ‘development projects'. But most of the reactions were at a superficial level, attempting to repair or fix what was considered inappropriate applications of classical development. This resulted in Western development being declared deceased and then at the same time being resuscitated. It became a zombie concept, dead and alive at the same time. Development, 2011, 54(4), (441–447) r 2011 Society for International Development 1011-6370/11 *www.sidint.net/development/* pp. 441-442.

^21^Sen, *Standard of living* p. 15.

^22^These observations come from my informal surveys. You are invited to conduct your own.

^23^A source that discusses gender differences in boredom: Vodanovich, S. J., and Kass, S. J. (1990). A Factor Analytic Study of the Boredom Proneness Scale. Journal of Personality Assessment, 55(1-2), 115–123. https://doi.org/10.1080/00223891.1990.9674051.

^24^Nussbaum 2011 p. 2.

^25^Nussbaum 2011 p. 21; Nussbaum 2000, 84.

^26^Sen *Standard*, “Adam Smith not only distanced his own approach from commodity fetishism and wealth maximization, he also showed the social nature of these relationships between commodities (and opulence), on the one hand, and capabilities (and achievements of living conditions), on the other.” p. 23 from *An Inquiry into the Nature and Causes of the Wealth of Nations* (1776; Everyman's edition, London: Home University Library, 1910), book V, ch. II, pp. 351-53. *https://www.earlymoderntexts.com/assets/pdfs/smith1759.pdf*. Nussbaum refers to Smith's Stoic thinking, see *Creating Capabilities*, 133.

^27^Adam Smith *Theory of Moral Sentiment* (1759): “No matter how selfish you think man is, it's obvious that there are some principles in his nature that give him...” [Sen dismisses the single-minded approach to earning, vs. commodity fetishism: “his approach goes back not only to Marx, but also to Adam Smith. In fact, despite the frequent claim that Adam Smith was mainly concerned with “wealth maximization,“ there is much evidence that he was deeply concerned with avoiding concentration on commodities (and wealth) as such, and keen on escaping the fetishism of which Marx spoke later.28 In fact, Adam Smith went well beyond the standard characterizations of living conditions and considered such functionings as not being “ashamed to appear in public,” and analyzed how the commodity requirements for this achievement - clothing, shoes, etc. - varied with social customs and cultural norms. [*An Inquiry into the Nature and Causes of the Wealth of Nations* (1776; Everyman's edition, London: Home University Library, 1910), book V, ch. II, pp. 351-53.] These customs and norms are, in their turn, influenced by the economic conditions of the respective societies. In analysing these relationships, Adam Smith not only distanced his own approach from commodity fetishism and wealth maximization, he also showed the social nature of these relationships between commodities (and opulence), on the one hand, and capabilities (and achievements of living conditions), on the other. The same capability of being able to appear in public without shame has variable demands on commodities and wealth, depending on the nature of the society in which one lives.” (*Standard of Living*, p. 23)].

^28^See exemplary debate in Charles Taylor, and Amy Gutmann, A. (1992). *Multiculturalism and “The Politics of Recognition”: an Essay*. Princeton University Press.

^29^Sen, “Culture and Development,” “Culture would have to figure among the end-based considerations in development analysis. But is the linkage entirely through the ends of development? What about the role of cultural factors as means of development? For example, cultural conditions can exert a strong influence on human behavior.” P.1.

^30^“It was a slow night,” is the policewoman's final comment that Trecia Reaves remembers from unexpected harassment inside a Boston Apartment.

^31^For support of the claim that Sen and Smith see pleasure as linked to dignity and autonomy: Smith, A. (1759). The theory of moral sentiments. Printed for A. Millar, and A. Kincaid and J. Bell, in Edinburgh.; Sen, A. (1992). Inequality reexamined. Russell Sage Foundation ; Harvard University Press.

^32^Standard of living p. 25.

^33^*Standard of living* p. 7, 10.

^34^Sen is not persuaded by Weber's well-known distinction between Catholic and Protestant varieties of capitalism.

^35^See Gosling and Taylor, The Greeks on Pleasure (1982), and M. C. Nussbaum, The Fragility of Goodness: Luck and Ethics in Greek Tragedy and Philosophy (Cambridge: Cambridge University Press, 1985).

^36^See: Kant, I. (1790). Critique of Judgment. Hackett Publishing.

^37^”Spieltrieb" refers to Friedrich Schiller's concept of play drive or play instinct. See: Friedrich Schiller. (2006). On the Aesthetic Education of Man, in a Series of Letters (Über die ästhetische Erziehung des Menschen). In German Idealism (p. 230). Edinburgh University Press. https://doi.org/10.3366/j.ctvxcrxbr.18

^38^Friedrich Schiller *Letters on the Aesthetic Education of Man* (original 1794, Letter IX). *Letters Upon The Aesthetic Education of Man (public-library.uk)*.

^39^Jacques Rancière's preferred term for the aesthetic effect is “disconnection” in the line of Shklovsky's “estrangement” with acknowledgment to Kant and to Schiller's disinterest. See “Art Is Going Elsewhere. And Politics Has to Catch It,” An Interview with Jacques Ranciére,” Sudeep Dasgupta, *Krisis, Journal for contemporary philosophy*, Issue 1, 2008 pp. 70-76. P. 74, 71. https://archive.krisis.eu/art-is-going-elsewhere-and-politics-has-to-catch-it-an-interview-with-jacques-rancie re/interview in *Krisis*.

^40^Kramer p.17.

^41^See: Bateson, G. (1972). Steps to an Ecology of Mind. Ballantine Books, Inc.

^42^For example, Lesley Caldwell, *Art, Creativity, Living* (London: Karnac Books, 2000), 16.

^43^D.W. Winnicot, “Playing: Its Theoretical Status in the Clinical Situation,” in *The International Journal of Psycho-Analysts*, vol. 49 (1968): 48. “As I look back over the papers that mark the development of my own thought and understanding I can see that my present interest in play in the relationship of trust that may develop . . . was always a feature of my consultative technique.”

^44^(Schiller, Letter XV) Carl Jung (1931): 46 kept the mantra in focus for Winnicott's readers: “man is completely human only when he is at play.” Quoted in Ellen Y. Siegelman, *Metaphor and Meaning in Psychotherapy* (New York: Guilford Press, 1993), 172.

^45^Winnicot, *Playing and Reality* (London: Tavistock Publications, 1971), 38. Cited in Siegelman, 172.

^46^Winnicott “Playing: Creative Activity and the Search for Self” (1971): 54-55. Play therapy manages to stay responsive to children's prompts. See, for example, Lawver, and Blankenship, K. (2008). “Play therapy: a case-based example of a nondirective approach.” *Psychiatry* (Edgmont (Pa.: Township)), 5(10), 24–28.

^47^Freud invokes the authority of Schiller to describe the opposition of the Ego and the Id. See Paul-Laurent Assoun, *Freud and Nietzsche* (London, Continuum Collection, 2002; originally in French, Presses Universitaires de France, 1980), 84. In 1908 Freud wrote: “Might we not say that every child at play behaves like a creative writer, in that he creates a world of his own, or, rather, rearranges the things of his world in a new way which pleases him?” Sigmund Freud, “Creative Writers and Daydreaming” (1908) in J. Strachey, ed., *The Standard Edition of the Complete Psychological Works of Sigmund Freud*, vol. 9 (London: Hogarth Press, 1955), 14.

^48^For more information on Melanie Klein's perspective on play, see her book Klein, M. (1975). Love, guilt, and reparation and other works, 1921-1945. Delacorte Press/S. Lawrence.

^49^Winnicott, *Playing and Reality*, 70.

^50^See David Rufo, “Math Hater: How One Child Overcame Her Math Anxiety Through Self-Administered Art Therapy,” *Art Education*, September 2017 n Volume 70, No. 5 pp 6-10. Rufo cites Brent Wilson, (1976). “Little Julian's impure drawings: Why children make art.” *Studies in Art Education, 17(2), 45-61*.

^51^Mihai Spariosu (in Dionysus Reborn: Play and the Aesthetic Dimension in Modern Philosophical and Scientific Discourse (Ithaca: Cornell University Press, 1989), 185.

^52^See Peter Beresford, (2002) “Thinking about ‘mental health': towards a social model,” *Journal of Mental Health*, 11(6), 581–584. *https://doi.org/10.1080/09638230020023921* See *Mental Health First Aid*; *http://empower.care* In Buenos Aires, the social model translates into artmaking, for example in the community focus of Proyecto Suma, *http://www.proyectosuma.org.ar/recuperacion-en-comunidad/* Another example historicizes clinical work focused on pathologies. See a documentary with a Foucaultian frame, by Francisco Díaz, “Desmontar la máquina / Dismantle the machine”.” *https://youtu.be/u8yAPiyPUso* And Vikram Patel's Projects In India, Mental Health Innovators: *https://www.mhinnovation.net/*; an interdisciplinary initiative at Harvard: *https://globalhealth.harvard.edu/domains/global-mental-health-harvard/*; and the new digital *http://empower.care/*.

^53^*Agrobiodiversity as Jazz Improvisation | ReVista (harvard.edu)* Karl Zimmerer, “Agrobiodiversity as Jazz Improvisation Foodscape: Change and Continuity,” *ReVista*, Fall 2016, *Volume XVI, Number 1* Sep 25, 2016.

^54^The case has been made, but needs more general adoption. See, for example, Deidre Heenan (2006). “Art as therapy: an effective way of promoting positive mental health?” *Disability and Society*, 21(2), 179–191. *https://doi.org/10.1080/09687590500498143*

^55^The following book explores the link between bipolar disorder and creativity including the lives and works of writers, artists, and musicians who have been diagnosed with bipolar disorder such as Vincent Van Gogh, Virgina Woolf, and Ludwig van Beethoven: Jamison, K. R. (1993). Touched with fire : manic-depressive illness and the artistic temperament. Free Press ; Maxwell Macmillan Canada ; Maxwell Macmillan International.

^56^References from Jose Falconi. *5 Famous Artists Who Used Art as Therapy (masterpeacebox.com)* “art as a means of self-expression and therapy is not a new concept. many famous artists used their art to work through everything from heartbreak and loneliness to joy and rage.” See also *Many Artists Suffered With A Mental Illness – Affinity Magazine* by Maariya Bhari, December 2, 2016. (*Affinity* is a teen-written and edited online magazine.) “art has always been a powerful outlet, dating back to Ludwig van Beethoven and Vincent van Gogh to Georgia O'Keeffe. Mental illnesses affects so many people today. All of the examples above who have exuded art from their very surroundings have battled mental illnesses and made peace with their demons while producing some of the most well known forms of art.”

^57^Viktor Frankl's important book, *Man's Search for Meaning* has evidently influenced the standard clinical connection between meaning and mental health. Originally published anonymously in German (1946), as *A Psychologist Experiences the Concentration Camp*, the English translation, *Man's Search for Meaning* (1959) became an international bestseller. (See *The Life of Viktor Frankl - The Viktor E. Frankl Institute of America % % (viktorfranklamerica.com)*.) For Frankl, the writing cure, logotherapy, meant producing purpose, not poetry. But survivors know a range of coping strategies. Some search for meaning; others distance themselves as observers, as artists. Here is Alfred Kantor's counterpoint from the same concentration camp, Terezin: “My commitment to drawing came out of a deep instinct of self-preservation and undoubtedly helped me to deny the unimaginable horrors of life at the time. By taking on the role of an ‘observer' I could at least for a few moments detach myself from what was going on in Auschwitz and was therefore better able to hold together the threads of sanity.” Alfred Kantor. (1987). *The book of Alfred Kantor: an artist's journal of the Holocaust*. Schocken Books. “Introduction,” no page no. See also policeman Winston Pingeon, traumatized by the January 6, 2022 insurrection who turns to painting: *https://www.bostonglobe.com/2023/01/05/arts/he-defended-capitol-democracy-itself-amid-insurrection-twoyears-later-he-paints-preserve-history-heal/?s_campaign=breakingnews:newsletter* Scholars do comment on the therapeutic effects of writing [see Koopman C., Ismailji T., and Holmes D. (2005). “The effects of expressive writing on pain, depression and Posttraumatic Stress Disorder symptoms in survivors of intimate partner violence.” *Journal of Health Psychology*, 10, 211–221.] – but many are skeptical if the writing doesn't make meaning. [See, Kaufman, J. C., and Sexton, J. D. (2006). “Why Doesn't the Writing Cure Help Poets?” *Review of General Psychology*, 10(3), 268–282. *https://doi.org/10.1037/1089-2680.10.3.268* p. 268: “there are two conflicting lines of thought about the creative writer and mental health. These two approaches seem to directly contradict each other. The first approach we call the mad writer. Images of Edgar Allan Poe, Emily Dickinson, or Sylvia Plath may dance through your head as you ponder the writer battling inner demons and torments. The second approach is that of writing as therapy. This concept encompasses the many benefits of creative writing.] But since poets seem to stay more afflicted than narrators, poets are “advised to adopt a narrative style,” to organize their confused thoughts into “a story with a beginning, middle, and end.” See more references in Christa Taylor, “Creativity and Mood Disorder: A Systematic Review and Meta-Analysis” *Perspectives on Psychological Science* Research article First published online September 21, 2017, *Volume 12, Issue 6* *https://journals.sagepub.com/doi/10.1177/1745691617699653* Consider John Dewey's.

^58^Joseph LeDoux, author of *Anxious: using the brain to understand and treat fear and anxiety*. Viking, an imprint of Penguin Random House (2015). is also a rock musician: “Together with Harold Koplewicz, a professor of child and adolescent psychiatry at NYU, and the support of NYU's administration, this institute will be dedicated to the study of emotions, especially fear and anxiety, in young brains – in both animals and children. We want to start teaching kids how to regulate their emotions. I also want to make emotion a university-wide integrative topic at NYU that can unify the arts and humanities (literature, history, visual and performing arts) and the applied disciplines (business, law) with the sciences.” See Jonathan Cott, Karen Rester, “Joseph LeDoux's heavy mental: The neuroscientist explains how music, emotion and memory shape our identities – and why he has donned a Stratocaster to keep the brain rollin' all night long.” *Salon*, July 25, 2007 *https://www.salon.com/2007/07/25/joseph_ledoux/*.

^59^An example of this loss appears in Lani Gerity's “Introduction” to Edith Kramer's collected papers. Kramer, a founder of art therapy, celebrates her teacher, Friedl Dicker – who taught children art in Terezin, before she and most of them were murdered – for illustrating “the sustaining and healing power of art.” Edith Krämer, and Gerity, Lani. A. (2000). *Art as Therapy: Collected Papers*. (London, Philadelphia, Jessica Kingsley Publishers.) p. 21. But Gerity wants more. She editorializes to feature meaning and reconciliation, along with hope and dreams: “teaching art to children in Terezin, helping them *keep hope and dreams alive*, and then not surviving Auschwitz herself. There is something in this chapter that teaches us about the possibility of *finding meaning in sorrow and suffering, and finally a strong sense of resolution and reconciliation*.” (my emphasis) Ibid, p. 11. Meaning is what makes Kaufman and Sexton (see above) prescribe prosaic writing. See also Pennebaker, and Graybeal, A. (2001). “Patterns of Natural Language Use: Disclosure, Personality, and Social Integration.” *Current Directions in Psychological Science : a Journal of the American Psychological Society*, 10(3), 90–93. *https://doi.org/10.1111/1467-8721.00123*; and Sloan, and Marx, B. (2004). “Taking pen to hand: Evaluating theories underlying the written disclosure paradigm.” *Clinical Psychology* (New York, N.Y.), 11(2), 121–137. *https://doi.org/10.1093/clipsy/bph062*

^60^For more on Augusto Boal's work and theories, refer to: Boal, A. (2000). Theater of the oppressed. London, UK: Pluto Press.

^61^Augusto Boal, (1995). *The Rainbow of Desire: the Boal Method of Theatre and Therapy*. (London: Routledge, 1995). P. 14. See also Alfred Kantor, op. cit. regarding the artist as distanced “observer.”

^62^Augusto Boal, (1995). *The Rainbow of Desire: the Boal Method of Theatre and Therapy*. (London: Routledge, 1995). “This movement forward in space and time is, in itself, therapeutic, since all therapy, before proposing the exercise of a choice, must consist of an inventory of possible alternatives. A process is therapeutic when it allows — and encourages — the patient to choose from several alternatives to the situation in which he finds himself, the situation which causes him unwanted suffering or unhappiness. In enabling, and indeed requiring, the patient to observe himself in action — since his own desire to show obliges him' both to see and to see himself — this theatrical process of recounting, in the present, and in front of witnesses ‘in solidarity', a story lived in the past, offers, in itself, an alternative.” P. 25.

^63^Some art therapists will dispute this privileging of process over product, pointing out that real art plumbs beyond the surface: “But this attitude of acceptance, which is essential in all therapy, must not dull the artist's capacity for discrimination. The teacher has to preserve her integrity as an artist in order to be able to distinguish between the fake and the genuine, between blocks and limitations, regressions and progress, superficial pretense, and genuine communication. . .” Edith Kramer, in Krämer, and Gerity, L. A. (2000). *Art as Therapy: Collected Papers*. (Jessica Kingsley Publishers.) p. 18. Philosophers in the tradition of ancient Greeks, value experience and reflection, not meaning. See Pierre Hadot, and Davidson, A. I. (1995). *Philosophy as a way of life: spiritual exercises from Socrates to Foucault*. Blackwell. See especially Chapter 8, “The Value of the Present.”

^64^“Contemporary developers of art therapy assessments have abandoned orthodox psychoanalytic approaches in favor of methods that emphasize the expressive potential of the tasks and materials (Cohen, Hammer, and Singer, 1988; Cox and Frame, 1993; Gantt and Tabone, 1997; Landgarten, 1993; Silver, 1978/1989).” *Handbook of Art Therapy*, Edited by CATHY A. MALCHIODI. (Guilford, 2003) p. 8.

^65^M.A. Maryam Hedayatia1, M.A. Mahmoud Khazaei “An Investigation of the relationship between depression, meaning in life and adult hope” *Procedia - Social and Behavioral Sciences Volume 114*, 21 February 2014, Pages 598-601 *Elseveir* [doi: 10.1016/j.sbspro.2013.12.753] “The experience of meaning in life has been recognized as an important contributor to health and well-being (Ryff and Singer, 1998; Wong and Fry, 1998). Indeed, in his classic work, Frankl (1963/1984) maintained that the need for meaning is a chronic, basic need (Heine, Travis, and Vohs, 2006). The importance of meaning in life is supported by research demonstrating its relations to a number of mental health variables such as depression, anxiety, hope, and life satisfaction (Mascaro and Rosen, 2005; Reker, Peacock, and Wong, 1987; Ryff, 1989; Steger and Frazier, 2005; Zika and Chamberlain, 1987, 1992). Steger and his colleague (2009) sees meaning in life as the manner in which people feel their lives have purpose, and how they comprehend their experiences.” Abstract: “Depression is one of the most common mental health problems in the world, so identifying variables that are related to it is very important. The purpose of the present study was to examine the relationship between *depression, meaning in life and hope [my emphasis]*. All 215 participants were students asked to complete the Beck Depression Inventory (1961), the Meaning in Life Questionnaire (MLQ) (in press) and Adult Hope Scale (AHS) (2002). The data of the study were analyzed by using the Pearson correlation coefficient. The results showed that there is a significant negative correlation between depression with meaning life (r = −0.479, p < .01), Presence meaning in life subscale (r = −0.511, p < .01) and Search meaning in life subscale (*r* = −0.286, *p* < 0.01). Also, the statistically significant correlation exist between depression and adult hope (*r* = −0.484, *p* < 0.01). It was found positive significant correlation between subscales of hope and meaning in life. Attention to these factors to minimize depression and to promote the society general health would be helpful. conference: Social and Behavioral Sciences 114 ( 2014 ) 598 – 601 1877-0428 © 2013 The Authors. Published by Elsevier Ltd. Selection and peer-review under responsibility of Academic World Education and Research Center. doi: 10.1016/j.sbspro.2013.12.753 ScienceDirect 4th World Conference on Psychology, Counseling and Guidance WCPCG-2013

^66^Osborn, T. L., Ndetei, D., Mutiso, V., Sacco, P. L., and Sommer, D. (2022, May 24). An Arts-Literacy Intervention for Adolescent Depression and Anxiety Symptoms: Outcomes of a Randomized Controlled Trial of Pre-Texts with Kenyan Adolescents. *https://doi.org/10.31219/osf.io/7cywv* [66] See the last 25 minutes of “Desmontar la máquina,” **.

^67^Hamlet, Act 2, Scene 2.

^68^Sen, (1985). *Commodities and Capabilities*. P 5 “It is possible for a person to have genuine advantages and still to ‘muff' them.

^69^Development as Freedom, Knopf 1999, p. 19 See also p. 77: “To insist that there should be only one homogeneous magnitude that we value is to reduce drastically the range of our evaluative reasoning. It is not, for example, to the credit of classical utilitarianism that it values only pleasure, without taking any direct interest in freedom, rights, creativity or actual living conditions.”

^70^Sen [1985 *Commodities and capabilities*] pp. 2-3: “This took the form of seeing utility as *satisfaction* or *happiness* (in line with classical utilitarianism), or as *desire-fulfilment* (in line with much of modern utilitarianism). But in much of modern p.3 economics ‘utility' serves other purposes too, standing for whatever the person maximizes (or can be seen as maximizing) or simply for the person's well-being or advantage no matter how that is judged. This loose usage has had a confounding influence on economic analysis. Mathematical exactness of formulation has proceeded hand in hand with remarkable inexactness of content.” On pp 21-22: “Our mental reactions to what we actually get and what we can sensibly expect to get may frequently involve compromises with a harsh reality. The destitute thrown into beggary, the vulnerable landless labourer precariously surviving at the edge of subsistence, the overworked domestic servant working round the clock, the subdued and subjugated housewife reconciled to her role and her fate, all tend to come to terms with their respective predicaments. The deprivations are suppressed and muffled in the scale of utilities (reflected by desire-fulfilment and happiness) by the necessity of endurance in uneventful survival.”

^71^Recently, there has been a discussion within the capability literature about whether capabilities and functionings should be limited to only encompass positively valued doings and beings — such as according to a normative theory, those that we have reason to value, or those which promote some ultimate normative aim, such as human dignity or agency — or whether capabilities and functionings are value-neutral concepts that encompass not only normatively positive but also negative and neutral doings and beings (Byskov 2020; Robeyns 2017).

^72^Sen's argument on the importance of flexibility in culturally conditioned values can be found in Sen, A. (1999). Development as freedom. Oxford (United Kingdom) Oxford Univ. Press.

^73^Amartya Sen *Commodities and Capabilities* 1985: p. 2 “The term utility does, of course, have meanings of its own, defined by utilitarians. It was used quite rigorously by utilitarian economists such as Edgeworth, Marshall, Pigou, Ramsey and Robertson. This took the form of seeing utility as *satisfaction* or *happiness* (in line with classical utilitarianism), or as *desire-fulfilment* (in line with much of modern utilitarianism). But in much of modern p.3 economics ‘utility' serves other purposes too, standing for whatever the person maximizes (or can be seen as maximizing) or simply for the person's well-being or advantage no matter how that is judged. This loose usage has had a confounding influence on economic analysis.”

^74^Pattnayak, S., and Stiglitz, J. E. (2012). The price of inequality: how today's divided society endangers our future [Review of The price of inequality: how today's divided society endangers our future]. International Studies, 49(3-4), −453. https://doi.org/10.1177/0020881714534542

^75^Carter, I., 2014, “Is the Capability Approach Paternalist?”, *Economics and Philosophy*, 30(1): 75–98. See also Dowding, K. and Van Hees, M., 2009, “Freedom of Choice”, in Anand, Pattanaik, and Puppe (eds.), *The Handbook of Rational and Social Choice*, Oxford: Oxford University Press, pp. 374–392.

^76^Amartya Sen. (1985). *Commodities and Capabilities*. North-Holland ; Sole distributors for the U.S.A. and Canada, Elsevier Science Pub. Co.: p.10. [https://archive.org/details/commoditiescapab0000sena/page/14/mode/2up?view=theater]

^77^An internet search “murder and suicide” brings up multiple and multiplying cases. For esample: “*Andrew Robinson fatally shot his wife, Linda Robinson, and 12-year-old son, Sebastian*, before turning the gun on himself inside the home on Porter Road early in the morning on Feb. 9…. Andrew Robinson was depressed and had trouble sleeping, witnesses told investigators. He'd sought help from professionals for physical and mental health problems, and had been given prescriptions for conditions — authorities didn't specify what conditions they were… The shooting was the third high-profile case of deadly family violence in Massachusetts this year, following killings in *Cohasset* and *Duxbury*. It rocked the community of Andover.” *Asher Klein* and *Kaitlin McKinley Becker* • Published February 22, 2023 *https://www.nbcboston.com/news/local/new-details-released-in-andover-murder-suicide-father-believed-tohave-called-911/2980225/#:$\sim$:text=Investigators%20have%20revealed%20more%20details*,*the%20morning %20on%20Feb.%209*. “APD investigating possible murder-suicide”Monday, Feb. 20, 2023, *ALBUQUERQUE –* APD's preliminary investigation into Sunday's homicide indicates a possible murder-suicide. Officers responded shortly after 2 p.m. on Sunday to a call about a possible overdose. Upon arrival at a home at 5327 Montgomery Blvd., N.E., officers discovered two people who died from gunshot wounds. The victim is identified as Isabella Bewley, 18. The second deceased individual is identified as William James, 21. The incident appears to be a domestic violence incident that resulted in a murder-suicide. The investigation is on-going.” Albuquerque Police Department, *https://www.cabq.gov/police/news/apd-investigating-possible-murder-suicide* “Prosecutors: Linden father kills wife, 2 children in apparent murder-suicide” BY LISA ROZNER UPDATED ON: FEBRUARY 20, 2023 / 6:25 PM / CBS NEW YORK” “LINDEN, N.J. – There is new information about an *unspeakable tragedy in New Jersey*.” *https://www.cbsnews.com/newyork/news/prosecutors-linden-father-kills-wife-2-children-in-apparent-murde~r-suicide/* John Diaz, Cory McCord (KHOU) Published: 5:48 AM CST February 19, 2023 Updated: 2:49 PM CST February 20, 2023 GALENA PARK, Texas — *Four people* were killed Saturday night in an *apparent murder-suicide in Galena Park*, according to *Sheriff Ed Gonzalez*. It happened around 10:30 p.m. at a home on *2nd Street near N. Main St. and Clinton Dr*. According to Gonzalez, a 12-year-old called the *Galena Park Police Department* and said she was sexually assaulted by her mother's boyfriend, who also shot people at the house. She told police that she grabbed her 1-year-old niece and ran to a neighbor's house for help when she got the opportunity. *https://www.khou.com/article/news/crime/houston-shooting-4-dead-murder-suicide/285-fa92f28d-30ae-4ce~d-89e2-b04276b16b2e* Married couple dead after apparent murder-suicide in ... - WPXI https://www.wpxi.com › news › local › 1-person-shot-c... 1 day ago — Emergency crews, including SWAT units, were stationed in the 400 block of Ashbury Court for reports of shots fired just after 11 a.m.. Police ... *Detroit police officers identified after murder-suicide in Livonia* *https://www.wxyz.com* ›* news › detroit-police-officers-… Kayla Ruble* The Detroit News “Two Detroit police officers were found dead in an apartment in Livonia on Sunday, according to law enforcement. Detroit police Chief James White said the bodies were discovered in the suburban apartment and that the officers' deaths appeared to have been a murder-suicide” *https://www.detroitnews.com/story/news/local/wayne-county/2023/02/19/detroit-police-officers-murder-sui* *cide-livonia/69921367007/* days ago — Livonia police identified the couple involved in Sunday's murder-suicide as Matthew Ethington II, 26, and Maria Martin, 22. Family reveals new details after murder-suicide in Waukee https://www.kcci.com › article › waukee-police-identify... days ago — Waukee police say 26-year-old Nelcybert Estafani Castillo Mata is the victim in a murder-suicide. Castillo Mata's family told KCCI she was a ... Police identify victim in Indianola murder-suicide case - KCCI https://www.kcci.com › article › police-identify-victim-i... 1 day ago — The victim is identified as 59-year-old Sheila Ann Kelly, who was in a domestic relationship with 56-year-old Jack Gordon Heiss Jr. He was also ...

^78^The concept of human capabilities was developed by economist and philosopher Amartya Sen and refers to a person's ability to lead a fulfilling and valuable life. See Sen, A. (1999). Development as freedom. Oxford (United Kingdom) Oxford Univ. Press.

^79^Standard of living p. 36 “I have tried to argue elsewhere that this may well be a good way to proceed, but I am less sure of this now.13 Being psychologically well-adjusted may not be a “material” functioning, but it is hard to claim that that achievement is of no intrinsic importance to one's standard of living. In fact, any achievement that is rooted in the life that one oneself leads (or can lead), rather than arising from other objectives, does have a claim to being directly relevant to one's standard of living.”

^80^This is the rhetorical question that Mayor Antanas Mockus of Bogotá, Colombia, would pose to skeptics.

^81^Viktor Shklovsky, “Art as Technique” 1917; “El arte como artificio” in the Spanish version. https://warwick.ac.uk/fac/arts/english/currentstudents/undergraduate/modules/fulllist/first/en122/lecturelist2015-16-2/shklovsky.pdf.

^82^See Raymond Williams, “Culture” in *Keywords*, Oxford UP, 1976.

^83^Sen standard of living 50.

^84^Stanford Encyclopedia: “Nussbaum passionately advocates that all people all over the world should be entitled, as a matter of justice, to threshold levels of all the capabilities on her list; but apart from mentioning that it is the governments' duties to guarantee these entitlements (2006: 70), she remains silent on the question who precisely should bear the burdens and responsibilities for realizing these capabilities. Yet as O'Neill (1996: chapter 5) has argued, questions of obligations and responsibilities should be central to any account of justice.”

^85^Subsequent studies confirmed these data (Boserup et al., 2020; Bradbury-Jones and Isham, 2020; Campbell, 2020; Leslie and Wilson, 2020; Miltimore, 2020; Mlambo-Ngcuka, 2020; Usher et al., 2020; World Health Organization, 2020; African News Agency, 2020).
